# Cytokine Changes With Effective Drug Therapy for Juvenile Myasthenia Gravis

**DOI:** 10.1155/jimr/3929690

**Published:** 2026-07-29

**Authors:** Yuanyuan Geng, Peng Liu, Min Jin, Guoyan Qi, Xiaoting Lin, Yousheng Zhang

**Affiliations:** ^1^ Shijiazhuang People’s Hospital, Shijiazhuang, Hebei, China; ^2^ Hebei Provincial Key Laboratory of Myasthenia Gravis, Shijiazhuang People’s Hospital, Shijiazhuang, Hebei, China; ^3^ Hebei Provincial Clinical Research Center for Myasthenia Gravis, Shijiazhuang People’s Hospital, Shijiazhuang, Hebei, China; ^4^ School of Basic Medicine, North China University of Science and Technology, Tangshan, Hebei, China, ncst.edu.cn; ^5^ Faculty of Medicine and Health, The University of Melbourne, Parkville, Melbourne 3010, Victoria, Australia, unimelb.edu.au

**Keywords:** cytokine, myasthenia gravis, signal pathway, treatment

## Abstract

**Objective:**

In order to investigate the therapeutic mechanism of Jianpiyiqi Granule in juvenile myasthenia gravis (JMG) and find the immune‐associated cytokines or signaling pathways targeted by this intervention.

**Methods:**

(1) We extracted JMG serum samples from the sample bank of the Hebei Provincial Key Laboratory of Myasthenia Gravis before and after treatment. (2) Using Olink Immune Response Panel detects serum cytokines. Data were collated and analyzed using statistical software. Differentially expressed proteins (DEPs) were visualized using heat maps, volcano plots, and other tools. Differentially expressed cytokines were analyzed using KEGG enrichment pathway analysis to identify the relevant signaling pathways they participate in.

**Results:**

(1) Twelve of 92 cytokines were found to be differentially expressed from 20 patients before and after treatment, namely AXIN1, CCL11, CCL13, CCL20, CCL25, CCL3, CD40, IL12B, S100A12, SIRT2, SLAMF1, and STAMBP (|log_2_FC| > 0.263; false discovery rate (FDR)‐adjusted *p*  < 0.05). IL12B was upregulated while AXIN1, CCL11, CCL13, CCL20, CCL25, CCL3, CD40, S100A12, SIRT2, SLAMF1 and STAMBP were downregulated after treatment. Notably, three of these—CCL11, IL12B, and STAMBP—showed stronger statistical evidence, with FDR‐adjusted *p*  < 0.01 and |log_2_FC| > 0.263. (2) The following pathways were obtained by KEGG enrichment analysis of 12 cytokines: cytokine–cytokine receptor interaction, viral protein interaction with cytokine and cytokine receptor, chemokine signaling pathway, Toll‐like receptor (TLR) signaling pathway, intestinal immune network for IgA production, IL‐17 signaling pathway, NF‐kappa B signaling pathway. (3) CCL11 and CCL20 were enriched in the IL‐17 signaling pathway. We used enzyme‐linked immunosorbent assay (ELISA) to retest additional serum samples before and after treatment from 12 JMG patients before and after treatment. The results showed a significant decrease in CCL11 and CCL20 in after‐treatment serum (*p*  < 0.05).

**Conclusions:**

Drug therapy for JMG revealed 12 differentially expressed cytokines. Due to the exploratory nature and sample volume constraints, only two candidate cytokines were validated by ELISA. We found that the cytokines CCL11 and CCL20—associated with the IL‐17 signaling pathway—were downregulated after treatment. The drug therapy may inhibit MG progression by reducing the expression of IL‐17‐associated cytokines. At the same time, the drug reduced the expression of CCL3, CCL13, CCL25, and CD40, which may play a role in the abnormal proliferation of thymocytes and chronic inflammatory response at the neuromuscular junction. Future studies with multiplex assays are needed to confirm the involvement of other differentially expressed cytokines, such as SIRT2 and AXIN1, which may contribute to disease mechanisms outside the identified pathways.

## 1. Introduction

Myasthenia gravis (MG) is an acquired autoimmune disease, mainly caused by the production of autoantibodies leading to the dysfunction of neuromuscular junction transmission [1, 2]. MG is divided into ocular‐MG and generalized‐MG based on the affected muscle groups. According to the age of onset, MG can be divided into juvenile‐MG (JMG) and adult‐MG. Epidemiological findings indicate that women aged 20–40 are more likely to develop MG than men, while men aged 50–70 have a higher incidence than women [3]. JMG in Asians is mostly ocular muscle weakness, and the peak age of onset is 0–5 years old [4–6]. At present, the treatment of MG primarily involves medication, and surgical removal of the thymus is performed in cases with thymic abnormalities. The treatment methods for adult‐MG are relatively mature, and many drugs can be applied clinically, including cholinesterase inhibitors, steroids, nonsteroidal immunosuppressive agents, new targeted drugs, and intravenous immunoglobulin (IVIg) and plasmapheresis (PE) therapy. The long‐term use of oral medications may lead to pharmacological dependence. Steroids and nonsteroidal immunosuppressive agents can cause liver and kidney damage, osteoporosis, bone marrow suppression, elevated blood sugar levels, and increased infection risks, with a high risk of relapse after discontinuation. Meanwhile, some side effects are obvious and irreversible after the application of steroids in adolescents [7]. Several Chinese herbal medicines (CHMs) have been used as an adjunct therapy for MG. Clinical studies indicate that certain CHM interventions may offer a favorable safety profile when used under professional guidance [8–10].

However, there is a lack of standardized research data and clinical drug treatment data on JMG. Considering the advantages of the CHM, it is more suitable for the treatment of JMG, which is mostly ocular muscle weakness and characterized by mild symptoms. We observed that Chinese herbal compound preparation (Jianpiyiqi Granule) demonstrated clinical efficacy in treating JMG at our center. However, the therapeutic effects vary after treatment with the same kind of drugs, which may be related to the changes in cytokines induced by the drugs in the body. In order to further study the mechanism of action of Jianpiyiqi Granule in JMG, we extracted serum samples from the sample bank of the Hebei Provincial Key Laboratory of Myasthenia Gravis before and after drug treatment and performed cytokine analysis.

## 2. Materials and Methods

### 2.1. Participants

From March 2023 to October 2023, JMG patients were treated at the Treatment Center of Myasthenia Gravis of Shijiazhuang People’s Hospital. The patient’s prognosis and basic clinical information (gender, age of onset, risk factors, disease course, medication usage, treatment plan, etc.) were collected and followed up for 1 year. Twenty JMG patients who had not received any medication (excluding oral cholinesterase inhibitors) before admission and showed an effective response after Jianpiyiqi Granule treatment were enrolled in this analysis. During the follow‐up period (12 months), patients need to continue taking Jianpiyiqi Granule orally.

### 2.2. Diagnostic Criteria

The diagnosis of MG refers to the “Chinese Guidelines for Diagnosis and Treatment of Myasthenia Gravis 2020” [11]. On the basis of typical clinical characteristics of fluctuating MG, at least one of the following criteria can be diagnosed: positive results in the neostigmine test, reduced low‐frequency repetitive electrical stimulation in electromyography (EMG), and positive results in serum acetylcholine receptor antibodies (AChR‐Ab).

The MG classification was based on the American Myasthenia Gravis Foundation (MGFA) classification, which is divided into Type I, IIa/IIb, IIIa/IIIb, IVa/IVb, and V [12].

The detection methods and result interpretations of AChR‐Ab are as follows: We used the RSR radioimmunoassay kit from the UK to detect AChR‐Ab: label α‐bungarotoxin with ^125^I to bind to the dual antigens of fetal/adult, precipitate the complex with antihuman IgG, and detect the radioactivity intensity by γ counting. The measured value is negative if the measured value is less than 0.5 nmol/L.

### 2.3. Inclusion Criteria

(1) The diagnosis of MG is clear, and the MGFA classification is type I. (2) Age of onset <18 years old. (3) Untreated before admission (excluding oral cholinesterase inhibitors), and the treatment plan is oral Jianpiyiqi Granule.

### 2.4. Exclusion Criteria

(1) Combination therapy with steroids or immunosuppressants before admission or during Jianpiyiqi Granule treatment. (2) Those who cannot tolerate Jianpiyiqi Granule treatment or are allergic to a certain component of Jianpiyiqi Granule. (3) Incomplete basic or follow‐up data.

### 2.5. Efficacy Evaluation

Jianpiyiqi Granule (Patent Number 2016101299575) is mainly composed of Radix Astragali Mongolici, Radix Salviae Miltiorrhizae, Rhizoma Atractylodis Macrocephalae, Radix Bupleuri Chinensis, Rhizoma Cimicifugae Foetidae, Radix Angelicae Sinensis, Radix Glycyrrhizae, Pericarpium Citri Reticulatae, Corni Fructus, Radix Polygoni Multiflori, Fructus Lycii, Radix Morindae Officinalis, Caulis Spatholobi, and so forth. (Benxi Chinese Drug Factory, Liaoning, China). Patients take the medicinal preparation twice daily in the morning and evening [13]. During the treatment period, the dosage can be decreased only after the clinical symptoms are completely relieved. Complete remission is defined as the absence of muscle weakness symptoms upon clinical examination by a clinician after the discontinuation of oral cholinesterase inhibitors. After achieving the state of complete remission, the original dose was maintained for 6 months. If there are no symptoms, reduce the dosing frequency to once a day and maintain it for 6 months, then change to once every 2 days until complete discontinuation of the drug.

The quantitative MG score (QMGS) and the MG muscular endurance score (MGMES) were used to quantify the severity of ocular muscle weakness [12, 14]. The ocular muscle score of MGMES includes the degree of eyelid occlusion of the pupil, upward gaze duration of the eyelids, ocular movement assessment, and diplopia (Supporting Information).

Following Jianpiyiqi Granule treatment, the QMGS decreased to 0 or by ≥2 points during cholinesterase inhibitor‐free follow‐up, meeting the predefined efficacy criteria. Conversely, all other cases were deemed treatment‐ineffective.

### 2.6. Serum Samples

Approximately 5 mL of peripheral venous blood was collected from each patient into a test tube before treatment began and during patient follow‐up. The centrifuge was set to 3000 rpm for 10 min; the serum was separated and stored in a −80°C freezer for further analysis. Serum samples were collected at 6:00 AM on the day before the treatment. The serum collected during the most recent follow‐up after the patient met the predefined efficacy criteria was used as the after‐treatment sample for testing.

### 2.7. Olink Analysis

Using Olink Immune Response Panel (Olink Proteomics AB, Uppsala, Sweden) detects serum cytokines according to the manufacturer’s instructions. This method allows for the simultaneous analysis of 92 analytes, with only 1 μL required for each sample. The detection results are expressed as normalized protein expression (NPX) values, which represent any unit on the log_2_ scale, with higher values indicating higher levels of cytokine expression.

### 2.8. Statistical Analysis

The measurement data were statistically analyzed using GraphPad Prism 9. The average values of the analyzed data were expressed as *x* ± s. The paired Wilcoxon test was used to compare the expression levels of 92 cytokines before and after treatment, and the raw *p*‐values were obtained. To control the risk of false positives caused by multiple comparisons, the two‐stage false discovery rate (FDR) method developed by Benjamini, Krieger, and Yekutieli was applied to control FDR < 0.05, and the FDR‐adjusted *p*‐value of each factor was calculated. The FDR‐adjusted *p*  < 0.05 was considered statistically significant.

### 2.9. Differentially Expressed Proteins (DEPs) Analysis

The median difference was used to represent the log_2_ (fold change [FC]) of each cytokine before and after the treatment. Differentially expressed cytokines were selected if the FC was <0.83 or >1.2 (|log_2_FC| > 0.263), and the FDR‐adjusted *p*  < 0.05. Statistically significant cytokines were visually presented through volcano plots, heat maps, bar charts, etc. The volcano plots, the heatmaps and KEGG enrichment analysis were created using Sangerbox Tools [15].

### 2.10. Ethics Approval and Consent to Participate

This study received approval from the Ethics Committee of Shijiazhuang People’s Hospital (Number [2022]027) and adhered to the principles outlined in the Declaration of Helsinki.

## 3. Results

### 3.1. Characteristics of the Patients

We summarized the basic clinical characteristics of the patients. The MGFA classification was type I, with a male‐to‐female ratio of 11:9. The course of the disease ranged from 7 days to 7 years. The median age of onset was 6 years (range: 1–11). There were 13 cases (65%) without obvious risk factors for disease onset. The positive rate of AChR‐Ab was 80%. The initial symptoms were fluctuating blepharoptosis. Thirteen patients (65%) had a history of oral cholinesterase inhibitors. The sampling interval ranged from 1 to 9 months, averaging 5.10 ± 2.43 months. Before treatment, the QMGS score ranged from 1 to 6, with a median of 3 and a mean of 3.1. The mean change in QMGS before and after treatment was 2.60 ± 1.31. The MGMES before treatment ranged from 1 to 13, with a median of 5.5 and a mean of 5.55. The mean change in MGMES before and after treatment was 3.75 ± 2.43 (Table 1).

**Table 1:** Baseline clinical characteristics of patients (*N* = 20).

Item	Outcome
MGFA classification	I
Gender (male/female)	11/9
Age of onset (years old)	
Median (range)	6 (1–11)
Risk factors, *n* (%)	7 (35)
Disease course	7 days – 7 years
AChR‐Ab positive, *n* (%)	16 (80)
Serum samples time interval (months)	
Range	1–9
Mean ± SD	5.10 ± 2.43
QMGS before the treatment	3.10 ± 1.17
QMGS before and after treatment	2..60 ± 1.31
MGMES before the treatment	5.55 ± 3.03
MGMES before and after the treatment	3.75 ± 2.43

It should be further noted that among the four patients with negative AChR‐Ab, further examination of muscle‐specific kinase antibodies (MuSK‐Ab) was also negative. However, in one patient, both the neostigmine test and electromyogram supported the MG diagnosis. Two patients only underwent the neostigmine test, and the results supported the MG diagnosis. One patient only had an electromyogram, and the results also supported the MG diagnosis.

### 3.2. Differences Between the Patients Before and After Treatment

We applied the Olink analysis software to detect the levels of 92 cytokines. Using the paired Wilcoxon test and multiple correction methods, 15 cytokines were found to be differentially expressed, namely AXIN1, CCL11, CCL13, CCL20, CCL25, CCL3, CD40, DNER, FLT3LG, IL12B, IL5, S100A12, SIRT2, SLAMF1, and STAMBP (FDR‐adjusted *p*  < 0.05). Among them, 12 cytokines satisfied the differential expression threshold (|log_2_FC| > 0.263; equivalent to FC < 0.83 or FC > 1.2) with statistical significance (FDR‐adjusted *p*  < 0.05), namely AXIN1, CCL11, CCL13, CCL20, CCL25, CCL3, CD40, IL12B, S100A12, SIRT2, SLAMF1, and STAMBP (Table 2). Notably, three of these—CCL11, IL12B, and STAMBP—showed stronger statistical evidence, with FDR‐adjusted *p*  < 0.01 and |log_2_FC| > 0.263.

**Table 2:** The list of differentially expressed cytokines.

Cytokines	Description	Raw *p*‐value	FDR‐adjusted *p*‐value	Fold change (FC, after vs. before)	Log_2_FC (after vs. before)
AXIN1	Axis inhibition protein 1	0.0056	0.0348	0.55	−0.85
CCL3	Chemokine ligand 3	0.0020	0.027	0.72	−0.48
CCL11	Chemokine ligand 11	0.0001	0.0027	0.77	−0.37
CCL13	Chemokine ligand 13	0.0056	0.0348	0.71	−0.49
CCL20	Chemokine ligand 20	0.0049	0.0348	0.67	−0.58
CCL25	Chemokine ligand 25	0.0014	0.0226	0.71	−0.50
CD40	Cluster of differentiation 40	0.0083	0.0447	0.76	−0.40
IL12B	Interleukin 12 subunit beta	0.0001	0.0027	1.57	0.65
S100A12	S100 calcium‐binding protein A12	0.0056	0.0348	0.61	−0.72
SIRT2	Sirtuin 2	0.0012	0.0226	0.35	−1.50
SLAMF1	Signaling lymphocytic activation molecule family member 1	0.0027	0.0312	0.77	−0.38
STAMBP	STAM binding protein	0.0001	0.0027	0.62	−0.68

**Figure 1:**
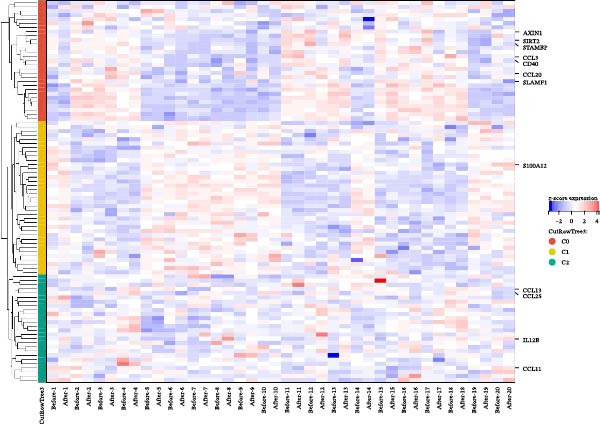
Heatmap of 92 cytokines. Correlation values are represented in color, with red indicating positive, blue negative, and darker colors indicating stronger correlations.

**Figure 2:**
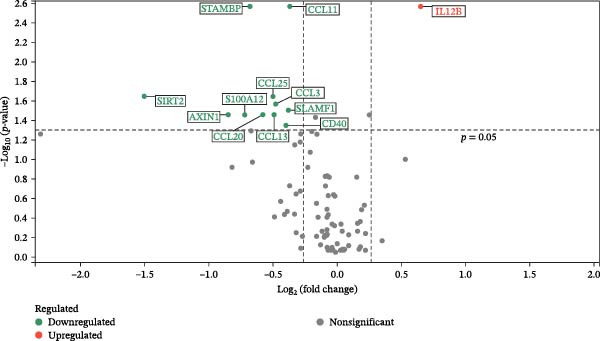
Volcano plots of 92 cytokines. The red circle represents upregulation, the green circle represents downregulation, and the gray circle represents nonsignificant cytokines, and 12 differentially expressed cytokines are annotated in the figure.

We made a heatmap of 92 cytokines (Figure 1), where red indicates high expression and blue indicates low expression. We also made volcano plots of 92 cytokines and annotated the differentially expressed cytokines (Figure 2). IL12B was upregulated, while AXIN1, CCL11, CCL13, CCL20, CCL25, CCL3, CD40, S100A12, SIRT2, SLAMF1, and STAMBP were downregulated.

### 3.3. DEPs Analysis

**Figure 3:**
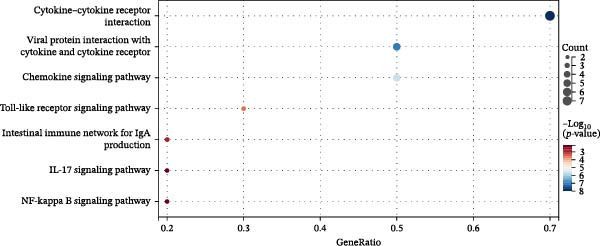
KEGG enrichment bubble plot of 12 differentially expressed cytokines. The bubble size (count) indicates the number of differentially expressed cytokines in each pathway. The color gradient represents the −log_10_ (FDR‐adjusted *p*‐value), where darker blue indicates greater significance. Pathways with a larger bubble and blue color are considered more significantly enriched and contain more differential cytokines.

The following pathways were obtained by KEGG enrichment analysis of 12 cytokines: cytokine–cytokine receptor interaction, viral protein interaction with cytokine and cytokine receptor, chemokine signaling pathway, Toll‐like receptor (TLR) signaling pathway, intestinal immune network for IgA production, IL‐17 signaling pathway, and NF‐kappa B (NF‐κB) signaling pathway (Figure 3).

**Figure 4:**
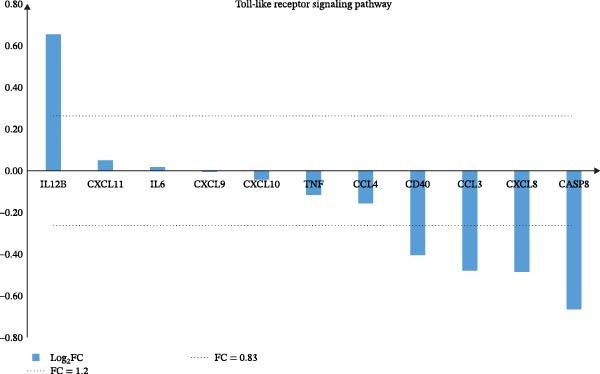
The list of cytokines associated with the Toll‐like receptor signaling pathway. All cytokines in the Toll‐like receptor signaling pathway are shown in the bar plot. The column represents the calculated value of log_2_FC, and the dotted line represents the threshold of |log_2_FC| = 0.263.

**Figure 5:**
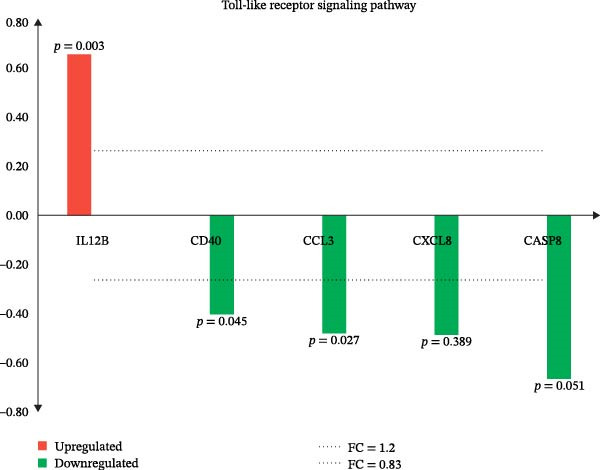
The list of differentially expressed cytokines associated with the Toll‐like receptor signaling pathway. Differentially expressed cytokines in the Toll‐like receptor signaling pathway are shown in the bar plot (|log_2_FC| > 0.263), the green column is downregulated, the red column is upregulated, and the *p*‐value marked is the FDR‐adjusted *p*‐value.

**Figure 6:**
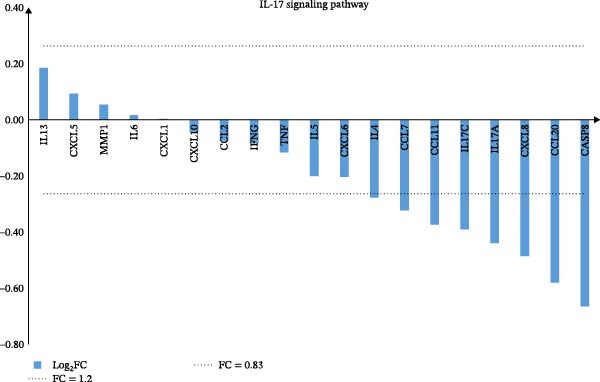
The list of cytokines associated with the IL‐17 signaling pathway. All cytokines in the IL‐17 signaling pathway are shown in the bar plot. The blue column represents the calculated value of log_2_FC, and the dotted line represents the threshold of |log_2_FC| = 0.263.

**Figure 7:**
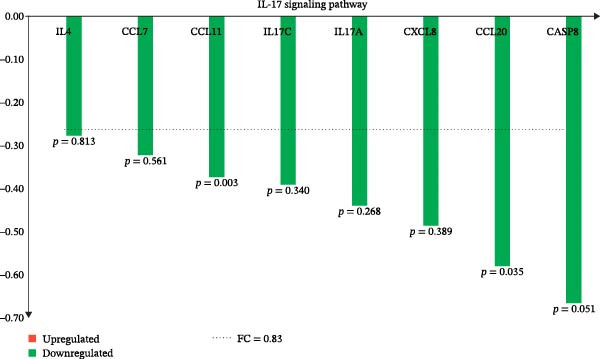
The list of differentially expressed cytokines associated with the IL‐17 signaling pathway. Differentially expressed cytokines in the IL‐17 signaling pathway are shown in the bar plot (|log_2_FC| > 0.263), the green column is downregulated, the red column is upregulated, and the *p*‐value marked is the FDR‐adjusted *p*‐value, and no upregulated cytokines were detected in this pathway.

We visualized the cytokines in the TLR signaling pathway among the 92 cytokines and found IL12B upregulated and four downregulated cytokines through bar charts (Figures 4 and 5). We also visualized all the cytokines in the IL‐17 signaling pathway and found that they were all downregulated through bar charts (Figures 6 and 7).

**Figure 8:**
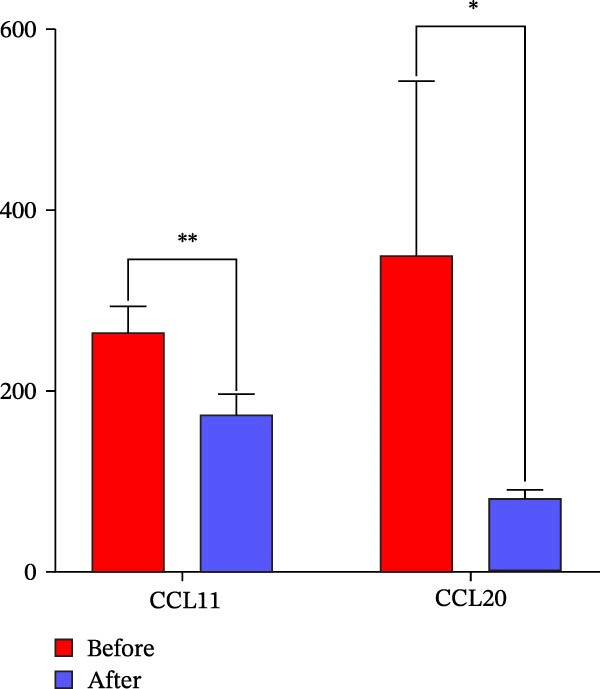
Enzyme‐linked immunosorbent assay.  ^∗^Represents *p* < 0.05 and  ^∗∗^represents *p* < 0.01; the red bars represent the pretreatment measurements and the blue bars represent the posttreatment measurements.

CCL11 and CCL20 were enriched in the IL‐17 signaling pathway. We used enzyme‐linked immunosorbent assay (ELISA) to retest additional serum samples before and after treatment from 12 JMG patients before and after treatment. The results showed a significant decrease in CCL11 and CCL20 in after‐treatment serum (*p*  < 0.05, Figure 8).

## 4. Discussion

MG is an acquired autoimmune disease that causes fluctuating muscle weakness. Other literature has preliminarily studied the regulation of serum cytokines by different immunosuppressive drugs, suggesting that these cytokines may be involved in the immune dysregulation associated with MG [16]. It is important for optimizing MG therapy to identify immune‐response‐related cytokines or signaling pathways involved in drug treatment during the disease process.

### 4.1. Differential Cytokines After Treatment

Even though the course of disease varies among patients, none had received any medication other than cholinesterase inhibitors before treatment at our hospital. Moreover, myasthenic symptoms were confined to the ocular muscles without progression. Therefore, we consider that these patients had a consistent baseline before receiving Jianpiyiqi Granule.

Using the Olink proteomics technology, we compared 92 cytokines in 20 JMG patients before and after treatment. Twelve differentially expressed cytokines were identified using a volcano plot with criteria of |log_2_FC| > 0.263 and FDR‐adjusted *p*  < 0.05. These cytokines may provide insights into the mechanisms of drug treatment for MG.

### 4.2. Pathway Enrichment Analysis

The “cytokine–cytokine receptor interaction” pathway [17], although the most significantly enriched, was considered uninformative because all measured items were cytokines. The “viral protein interaction with cytokine and cytokine receptor” pathway—which enriches CCL3, CCL11, CCL13, CCL20, and CCL25—has not been specifically linked to MG in the literature, although viral infection is generally believed to induce or exacerbate MG [18]. The “chemokine signaling pathway” regulates immune cell migration and function; many cytokines belong to this chemokine family [19]. These pathways have little reference to this study of drug‐associated cytokine‐mediated immune signaling pathways. Instead, we identified four KEGG pathways that are significantly associated with MG and are relevant to our findings (Table 3).

**Table 3:** Four KEGG pathways.

Pathway (KEGG ID)	Enriched cytokines	*q*‐Value
Toll‐like receptor (TLR) signaling pathway (hsa04620)	CCL3, CD40, IL12B	0.003
Intestinal immune network for IgA production (hsa04672)	CCL25, CD40	0.010
IL‐17 signaling pathway (hsa04657)	CCL11, CCL20	0.026
NF‐κB signaling pathway (hsa04064)	CCL13, CD40	0.026

#### 4.2.1. TLR Signaling Pathway

The TLR signaling pathway is a nonspecific immune pathway that initiates inflammatory responses through TLR activation and contributes to the pathogenesis of various autoimmune diseases. Given the presence of TLRs in the thymus, this pathway is implicated in MG development [20]. However, because both upregulated and downregulated cytokines were observed after drug treatment, we cannot definitively conclude whether Jianpiyiqi Granule activates or inhibits this pathway.

CD40 is a costimulatory molecule expressed on antigen‐presenting cells. Its interaction with CD40 ligand (CD40L) on activated T cells regulates B‐cell proliferation and differentiation. In MG, activation of the CD40‐CD40L axis promotes autoreactive B‐cell maturation and production of pathogenic autoantibodies, including AChR‐Ab and MuSK‐Ab [21]. The literature linking inflammatory cytokines and MG has also reported that CD40L expression positively correlates with MG [22]. In our study, CD40 signaling was suppressed in the TLR pathway, the intestinal immune network for IgA production, and the NF‐κB pathway. This suggests that Jianpiyiqi Granule may alleviate MG by modulating gut‐resident T and B lymphocytes. Supporting this, modified Buzhong Yiqi decoction has been shown to relieve MG symptoms by regulating intestinal flora and reducing serum IL‐17 levels [23].

#### 4.2.2. NF‐κB Pathway

Multiple studies have demonstrated that NF‐κB inhibition reduces the proportion of Th17 cells while increasing the proportion of Treg cells, thereby improving the Th1/Th2 and Th17/Treg imbalances and alleviating MG symptoms [24]. Our findings are consistent with this: downregulation of CCL13 and CD40 within the NF‐κB pathway aligns with clinical improvement.

CCL13, also known as MCP‐4, is a chemokine that plays an important role in inflammatory responses and immune diseases, especially chronic inflammatory diseases. Previous studies have shown that serum MCP‐4 decreases in effectively treated systemic lupus erythematosus patients [25], and inhibiting MCP‐4 reduces tumor cell invasiveness [26]. In our cohort, patients who showed significant symptom improvement after Jianpiyiqi Granule also exhibited decreased CCL13 levels.

#### 4.2.3. IL‐17 Signaling Pathway

The IL‐17 signaling pathway is associated with autoimmune diseases and is enriched in CCL11 and CCL20. IL‐17 is mainly produced by Th17 cells. Although IL‐17 does not directly induce AChR antibody production by plasma cells, it is involved in chronic inflammation at the neuromuscular junction, MG pathogenesis, and drug mechanisms. For example, experimental autoimmune MG mice with IL‐17 deficiency exhibited halted disease progression and produced lower levels of AChR‐Ab [27]. TLR signals can promote Th17 differentiation and suppress Treg function via NF‐κB activation, disrupting immune tolerance and activating the complement pathway to form membrane attack complexes that damage the neuromuscular junction [28]. In a study on the signal pathways of CHM in the treatment of MG based on network pharmacology, it was also found that MG was mainly treated through multiple targets and pathways such as the IL‐17 signaling pathway and Th17 cell differentiation [29].

CCL25 can drive the migration of γδT cells that secrete IL‐17 and produce inflammatory responses [30]. As early as 2013, it was proposed that abnormal proliferation of thymocytes in children with MG was related to the overexpression of CCL25 [31]. The downregulation of CCL25 observed in our study, therefore, suggests a potential mechanism by which Jianpiyiqi Granule may reduce IL‐17‐mediated inflammation.

In conclusion, the IL‐17 signaling pathway and Th17 cells interact with the TLR signaling pathway and the NF‐κB pathway. The IL‐17 signaling pathway may be a key mechanism underlying the therapeutic effect of Jianpiyiqi Granule.

### 4.3. Correlation With Clinical Severity

In addition to upregulation of IL12B, we observed downregulation of CCL3, CCL11, CCL13, CCL20, CCL25, and CD40, indicating that the drug inhibited multiple signaling pathways associated with MG. International studies have analyzed correlations between cytokines and the severity of MG, revealing that the expression levels of CCL3 and IL12B were significantly higher in patients with MG compared to healthy individuals. Furthermore, CCL3 and IL12B exhibited negative correlations with the MG activities of daily living scale (MG‐ADL), while IL‐17 showed positive correlations with MG‐ADL [32, 33].

### 4.4. Limitations

Several limitations should be acknowledged. First, the lack of a healthy control group is a major limitation. Although the self‐controlled before‐after design effectively evaluates treatment effects, it cannot determine whether cytokine levels return to a normal physiological state after treatment. Future studies should include age‐ and sex‐matched healthy controls to verify whether the identified differential factors represent true biomarkers of disease amelioration.

Second, the small sample size (*n* = 20), wide variation in disease duration, and failure to exclude confounding factors such as infections may have obscured other clinically relevant cytokines and precluded deeper mechanistic analyses.

Third, due to the exploratory nature of this study and limited sample volumes, only two candidate cytokines (CCL11 and CCL20) were validated by an ELISA. These two cytokines were selected because they are key components of the IL‐17 signaling pathway, which may serve as a central mechanism, and both showed consistent downregulation with high statistical significance. Future studies using multiplex assays are needed to confirm the involvement of other differentially expressed cytokines, such as SIRT2 and AXIN1, which were not directly within the TLR/IL‐17 pathways but may play other roles.

Finally, it should be noted that the safety evidence for CHM in MG is primarily derived from small‐sample or observational studies, and the risk of adverse events may vary across different Chinese herbal compound preparations. Therefore, the conclusion of “low toxicity” applies only to the specific preparations and treatment regimens reported in the cited literature and cannot be generalized to all CHM.

This study only included effective patients; therefore, the observed cytokine changes may not be generalizable to ineffective patients. Future studies should include both effective and ineffective groups to identify potential predictors of treatment.

## 5. Conclusion

Drug therapy for JMG revealed 12 differentially expressed cytokines. Due to the exploratory nature and sample volume constraints, only two candidate cytokines were validated by ELISA. We found that the cytokines CCL11 and CCL20—associated with the IL‐17 signaling pathway—were downregulated after treatment. Drug therapy may inhibit MG progression by reducing the expression of IL‐17‐associated cytokines. At the same time, the drug reduced the expression of CCL3, CCL13, CCL25, and CD40, which may play a role in the abnormal proliferation of thymocytes and chronic inflammatory response at the neuromuscular junction. Future studies with multiplex assays are needed to confirm the involvement of other differentially expressed cytokines, such as SIRT2 and AXIN1, which may contribute to disease mechanisms outside the identified pathways.

## Author Contributions

Data curation, investigation, writing – original draft: Yuanyuan Geng. Formal analysis: Yuanyuan Geng, Min Jin, Xiaoting Lin, and Yousheng Zhang. Project administration: Peng Liu and Guoyan Qi. Writing – review and editing: Yuanyuan Geng, Peng Liu, Min Jin, Guoyan Qi, and Xiaoting Lin.

## Funding

This study was supported by the National Natural Science Foundation of China (Grant 82274582), the Central Guidance for Local Scientific and Technological Development Funding Projects (Grant 246Z7706G), and the Key Laboratory Construction Subsidy Fund Project (Grant 236790017H). The principal funding recipient is Guoyan Qi.

## Disclosure

The manuscript is approved by all authors for publication.

## Ethics Statement

Ethical approval was granted by the Ethics Committee of Shijiazhuang People’s Hospital. We confirm that we have read the position on issues involved in ethical publication and affirm that this article is consistent with those guidelines.

## Conflicts of Interest

The authors declare no conflicts of interest.

## Supporting Information

Additional supporting information can be found online in the Supporting Information section.

## Supporting information


**Supporting Information** This Supporting Information contains additional data that support the findings reported in the main manuscript. Supporting Information details the items and criteria for the ocular muscle score of the myasthenia gravis muscular endurance score.

## Data Availability

The data that support the findings of this study are available upon request from the corresponding author. The data are not publicly available due to privacy or ethical restrictions.
