# Supplementary material for: Cytokine Changes With Effective Drug Therapy for Juvenile Myasthenia Gravis
Source: J Immunol Res. 2026 Jul 29;2026:3929690. doi: 10.1155/jimr/3929690 (PMC13420311; doi:10.1155/jimr/3929690)
Supplement: Supplementary file 1 — Supporting Information This Supporting Information contains additional data that support the findings reported in the main manuscript. Supporting Information details the items and criteria for the ocular muscle score of the myasthenia gravis muscular endurance score. [file JIMR-2026-3929690-s001.docx]

**Supplementary material 1**

**Myasthenia gravis muscular endurance score**

| muscle groups | Scoring items | normal | low-grade | moderate | severe |
| --- | --- | --- | --- | --- | --- |
| musculus ocularis | The upper eyelid covers the pupil (Clock position, Score separately on the left and right sides) | 11~1 (0 points) | 10~2 (1 point) | 9~3 (2 points) | Below 8~4 (3 points) |
|  | upward gaze (Record the time when the eyelids droop to 9-3, Score separately on the left and right sides) | Above 60s (0 points) | 31~60s (1 point) | 1~30s (2 points) | 0 (3 points) |
|  | Eye abduction with white exposure and adduction with white exposure, diplopia (Score separately on the left and right sides) | ≤2mm (0 points) | 3~7mm or diplopia (1 point) | 8~12mm (2 points) | >12mm (3 points) |
|  | Eyeball up and down vision | normal (0 points) | The upper and lower eyes of the binocular are not at the same level (1 point) | The two eyes are not at the same level when looking at eye level (2 points) | Fixed eyes, unable to move (3 points) |
